# Functionalization of Cellulose-Based Hydrogels with Bi-Functional Fusion Proteins Containing Carbohydrate-Binding Modules

**DOI:** 10.3390/ma14123175

**Published:** 2021-06-09

**Authors:** Mariana Barbosa, Hélvio Simões, Duarte Miguel F. Prazeres

**Affiliations:** 1 iBB—Institute for Bioengineering and Biosciences and Department of Bioengineering, Instituto Superior Técnico, Universidade de Lisboa, Av. Rovisco Pais, 1049-001 Lisboa, Portugal; helvio.simoes@tecnico.ulisboa.pt (H.S.); miguelprazeres@tecnico.ulisboa.pt (D.M.F.P.); 2 Associate Laboratory i4HB—Institute for Health and Bioeconomy at Instituto Superior Técnico, Universidade de Lisboa, Av. Rovisco Pais, 1049-001 Lisboa, Portugal

**Keywords:** biomolecular recognition, carbohydrate-binding module, cellulose, hydrogel, ionic liquid

## Abstract

Materials with novel and enhanced functionalities can be obtained by modifying cellulose with a range of biomolecules. This functionalization can deliver tailored cellulose-based materials with enhanced physical and chemical properties and control of biological interactions that match specific applications. One of the foundations for the success of such biomaterials is to efficiently control the capacity to combine relevant biomolecules into cellulose materials in such a way that the desired functionality is attained. In this context, our main goal was to develop bi-functional biomolecular constructs for the precise modification of cellulose hydrogels with bioactive molecules of interest. The main idea was to use biomolecular engineering techniques to generate and purify different recombinant fusions of carbohydrate binding modules (CBMs) with significant biological entities. Specifically, CBM-based fusions were designed to enable the bridging of proteins or oligonucleotides with cellulose hydrogels. The work focused on constructs that combine a family 3 CBM derived from the cellulosomal-scaffolding protein A from *Clostridium thermocellum* (CBM3) with the following: (i) an N-terminal green fluorescent protein (GFP) domain (GFP-CBM3); (ii) a double Z domain that recognizes IgG antibodies; and (iii) a C-terminal cysteine (CBM3C). The ability of the CBM fusions to bind and/or anchor their counterparts onto the surface of cellulose hydrogels was evaluated with pull-down assays. Capture of GFP-CBM3 by cellulose was first demonstrated qualitatively by fluorescence microscopy. The binding of the fusion proteins, the capture of antibodies (by ZZ-CBM3), and the grafting of an oligonucleotide (to CBM3C) were successfully demonstrated. The bioactive cellulose platform described here enables the precise anchoring of different biomolecules onto cellulose hydrogels and could contribute significatively to the development of advanced medical diagnostic sensors or specialized biomaterials, among others.

## 1. Introduction

New functional cellulose-based materials can be obtained through the assembly of bioactive molecules that enhance the existing properties or add novel potentialities [1]. Such matrices have the potential to be applied in a great number of different fields, ranging from functional textiles [2], smart packaging [3], and biomanufacturing [4] to biosensing [5] and advanced drug delivery systems [6].

Cellulose is the most abundant natural biopolymer on Earth and, accordingly, one of the most important biomass resources [7]. This biopolymer forms highly crystalline structures with elongated stiff chain packing due to the β(1,4)-linked glycosidic arrangement of glucose repeating units and acts as a structural material in biological systems [7,8]. Much of the biomedical focus on the use of cellulosic materials as scaffolds in tissue engineering is due to their biocompatibility and the relative ease with which they can be tuned to adjust mechanical properties and introduce several surface modifications [9]. The application of cellulose can be extended to soft and plasticized materials by incorporating some moieties among the cellulose chains that relax its stiff crystalline structure. For instance, cellulosic flexible materials have been successfully fabricated by adding plasticizers such as glycerin [10]. In addition, different swelling methodologies for cellulose have been used, yielding softer materials, such as in the case of hydrogels [11]. Cellulose hydrogels can be prepared from cellulose solutions by forming a hydrogen-bonded network through physical crosslinking via the abundant hydroxyl groups in the molecule. However, cellulose is extremely difficult to dissolve in water and most common organic solvents. Alternatively, bacterial cellulose is a strong candidate for the fabrication of cellulose-based hydrogels since certain bacterial species possess the ability to create pure cellulose hydrogels [12]. Additionally, cellulose acetate, a well-known derivative of cellulose produced by acetylation of native cellulose, has a much less crystalline structure, and thus exhibits better solubility in conventional solvents such as acetone [13,14]. 

New solvents, such as N-methylmorpholine-N-oxide (NMMO), alkali/urea (or thiourea) aqueous systems, and ionic liquids (ILs), have emerged has effective solvents for cellulose, opening up a greater range of prospects for the preparation of cellulose hydrogels. Since 1-*N*-butyl-3-methylimidazolium chloride ([BMIM][Cl]) was found to efficiently dissolve up to 25 wt.% cellulose [15], ILs have been widely exploited as powerful solvents for cellulose [16]. ILs are molten salts that are liquid at temperatures below the boiling point of water [17]. In this context, ILs, e.g., [BMIM][Cl], 1-ethyl-3-methylimidazolium chloride ([EMIM][Cl]), 1-*N*-butyl-2,3-dimethylimidazolium chloride ([BDMIM][Cl]), 1-*N*-butyl-3-methylimidazolium acetate ([BMIM][Ac]) and 1-ethyl-3-methylimidazolium acetate ([EMIM][Ac]), are particularly interesting, given their ability to form flexible cellulose films, through ion gel formation, with thermoplasticity and thermal processability without requiring previous cellulose processing such as acylation [18]. For instance, previous studies have reported the formation of a cellulose-based ion gel with [BMIM][Cl] by standing a cellulose/[BMIM][Cl] solution at room temperature (RT) [19,20]. Likewise, Satani et al. [21] obtained cellulose hydrogels with a wide range of concentrations using a [BMIM][Ac]/DMSO mixed solution. They formed the hydrogels without using a crosslinking agent by casting the cellulose solutions into molds and immersing them in deionized water to replace the [BMIM][ Ac]/DMSO solution with water [21]. In another study, cellulose flat sheet membranes were fabricated by phase inversion using [EMIM][Ac] and a mixed solution of [EMIM][Ac] and DMSO [19]. 

The growing demand for sustainable functional biomaterials can be partially met by adding relevant functionalities to cellulose. Cellulose-derived materials can be engineered by chemical modification or physical adsorption [9]. However, alternatives to these conventional modification methodologies can be developed by exploring the specific recognition properties of proteins. Carbohydrate binding modules (CBMs) are modular proteins with high binding specificity towards carbohydrates that could be used in this context. Particularly, type A CBMs display a planar carbohydrate-binding interface that is adapted to bind to the crystalline surface of the microfibrils in cellulose-based materials. Hence, the use of CBMs is a simple way to direct bioactive molecules to cellulose matrices, which avoids the use of more complex chemical grafting procedures or harsh chemical environments. Furthermore, the binding of such CBMs to cellulose is highly specific and stable. Indeed, CBMs can only be removed from cellulose surfaces by using denaturant agents [22,23]. In addition, given their extensive repertoire in nature, CBMs can be specifically chosen so that their binding features (e.g., optimal pH, temperature and ionic strength) correctly match the requisites of the final application. Indeed, it has already been demonstrated that different CBMs have specific substrate affinity and are able to recognize different crystalline, amorphous, soluble, or non-soluble polysaccharides [24,25]. 

In connection with the above, we have been working on the design of bi-functional recombinant fusion proteins of the CBM-X type by joining two genes, one coding for a CBM and the other coding for a protein X. This protein X may further possess affinity to a specific partner that could be later incorporated in or captured by the cellulose hydrogel. Many successful biorecognition systems are described in the literature that could be explored in the context of this cellulose:CBM-X:partner molecular architecture, including the FLAG tag:IgG [26,27], avidin:biotin [28,29], PDZ domain:peptide [30,31], and ZZ:IgG [32,33,34,35] systems. In another example published in the literature, a fusion of a CBM with lysozyme was designed and used to develop antimicrobial cellulosic wound dressing materials [36]. CBMs could thus be fused to a wide range of different biomolecules, depending on the desired final application [36,37,38,39]. Our group used biomolecular recognition based on CBMs to develop molecular paper-based devices [37] and to anchor gold nanoparticles (AuNP) on cellulose substrates [38]. The first element of the recombinant construct used therein was a CBM-X fusion that combined a family III CBM (CBM3) from the cellulosomal-scaffolding protein A from *Clostridium thermocellum* (*C. thermocellum*) with a double Z domain, which is an engineered variant of the consensus domain B of the staphylococcal protein A. The CBM part of the fusion bonded specifically to cellulose, whereas the ZZ domain was available to capture IgG antibodies via their Fc region. The second recognition element was an anti-biotin antibody, which was anchored onto cellulose via the ZZ-CBM fusion. This antibody was subsequently used to capture biotin-labeled AuNPs [38] or biotinylated oligonucleotides, previously hybridized with a complementary fluorescein-labeled DNA probe, giving rise to fluorescent signals [37]. The later immobilization of DNA strands on cellulose is extremely relevant for the development of paper-based molecular diagnostics [37] for the specific and sensitive detection of DNA or RNA sequences [33]. In the present study, other CBM3-based fusions were evaluated in addition to the ZZ-CBM fusion. Moreover, we evaluated the binding affinity of the bi-functional proteins onto a distinct cellulose material from the one described by Prazeres and colleagues, namely a cellulose-based hydrogel. Regarding the material, we also tested three hydrogels synthesized with different amounts of cellulose to determine the effect of cellulose concentration on CBM binding. Our goal was to prove that a bioactive platform can be developed that combines cellulose-based hydrogel scaffolds with fusions of carbohydrate binding modules to anchor different molecules. Further, we envisage the use of this platform in different biomedical applications that go beyond molecular diagnostics.

**Figure 1:**
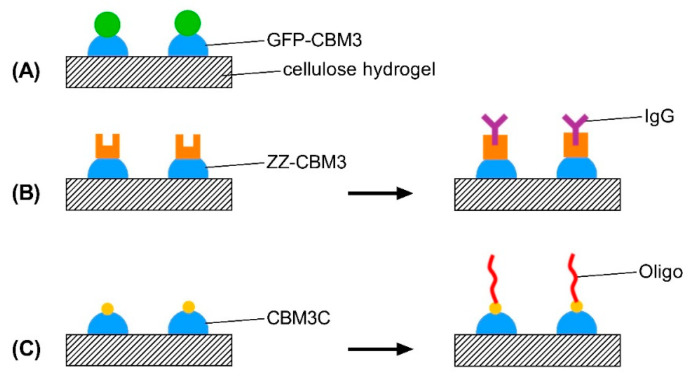
Schematic representation of the strategy used to functionalize cellulose hydrogels with: (**A**) a green fluorescent protein; (**B**) IgG; and (**C**) oligonucleotides. (**A**) A GFP-CBM3 fusion is used. (**B**) A ZZ-CBM3 fusion is first anchored on the hydrogels and then used to capture IgG. (**C**) A CBM3 with a C-terminal cysteine is pre-anchored on the hydrogels, which is then fused to a maleimide-terminated oligonucleotide via the formation of a covalent bond.

In the current proof-of-concept work, we present a straightforward biorecognition method to modify cellulose-based hydrogels. Specifically, we explore the properties of CBM3 from *C. thermocellum* to functionalize cellulose hydrogels with a green fluorescent protein, antibodies, and oligonucleotides (see Figure 1).

CBM3 binds strongly and specifically to crystalline cellulose fibrils due to a characteristic planar linear strip of aromatic and polar residues (namely, tryptophan, arginine, histidine, aspartic acid, and tyrosine), located in one of the faces of its nine-strand β-sandwich jelly roll structure [35]. The methodology allows an expedite functionalization of cellulose hydrogels under mild biological conditions without requiring complex chemical grafting procedures. Altogether, this paper aims to provide a proof-of-concept that not only is the CBM portion of the bi-functional protein able to bind to cellulose hydrogels but also that the fusion partner is still active, even after immobilization, and can successfully capture its counterpart. The long-term goal is to use such cellulose-based hydrogels to develop advanced tissue engineering materials and molecular biosensors for healthcare diagnostics. However, rather than developing a specific application, this study focused on the design, construction, and testing of a range of CBM-based molecular constructs that can be handled as ready-to-use protein-based platforms for distinct applications.

## 2. Materials and Methods

### 2.1. Materials

[EMIM][Ac] (>95% purity) from TCI Europe (Zwijndrecht, Belgium) was used to dissolve cellulose. Dimethyl sulfoxide (DMSO) was purchased from Sigma. The degree of polymerization of cellulose directly affects its solubility and is greatly influenced by the source of extraction [40]. Here, microcrystalline cellulose (MCC) with a 51 μm average particle size (Sigma), which is widely used for dissolution experiments, was chosen. The MCC powder was dried at 60 °C for 72 h prior to use. Human normal immunoglobulin (165 mg/mL, IgG) with a purity of at least 95 % was obtained from Octapharma (Manchester, Lancashire).

A 12 nt long oligonucleotide 5′-TTGAAGTCGAGG-3′ (DN3) containing sequences from the genome of the dengue virus was designed with a terminal 5′ amino-serinol and obtained from STAB VIDA (Oeiras, Portugal). The sulfosuccinimidyl 4-(N-maleimidomethyl) cyclohexane-1-carboxylate (sSMCC) crosslinker was obtained from Thermo Scientific^TM^.

All protein dilutions were prepared in Tris-Saline Tween (TST) buffer (50 mM Tris buffer pH 7.6, 150 mM NaCl, 0.05% *v*/*v* Tween 20), unless stated otherwise. Phosphate-buffered saline (PBS; 10 mM phosphate pH 7.2, 150 mM NaCl) was used in the to dilute the oligonucleotide. Type 1 water (resistivity higher than 18 MΩ-cm, conductivity lower than 0.056 µS/cm, and less than 50 ppb of total organic carbons) obtained with a Milli-Q purification system (Merck-Millipore, Portugal) was used in all buffers.

### 2.2. Preparation of Cellulose-Based Hydrogels

The preparation of cellulose hydrogels was conducted as follows: cellulose dissolution, molding, and gelation by solvent displacement. In the dissolution phase, specific amounts of MCC powder were added to 2 g of DMSO in a 20 mL vial and swelled for 15 min, under magnetic agitation. Then, [EMIM][Ac], previously dried at 100 °C for 3 h in a vacuum oven, was added to the swelled cellulose and the solution was stirred at 25 °C for 24 h. A 3:2 final ratio (weight) of [EMIM][Ac]:DMSO was used as recommended in previous studies [21,41]. Solutions with three different cellulose contents were obtained (Table 1), which were then soaked in 5 mL of Type 1 water [42] for 24 h, yielding clear hydrogels. These were then removed, washed five times with Type 1 water, and stored in ethanol solution to prevent contamination.

### 2.3. Surface Morphology Analysis of the Cellulose-Based Hydrogels

Scanning electron microscopy (SEM) was used to image the surface of the hydrogels. Micrographs of the test samples were taken using a high-resolution analytical SEM Hitachi S2400 with Bruker light elements EDS detector. Electron beam intensity of 20 kV (accelerating voltage) and different magnifications were used. To increase the surface conductivity, samples were pre-coated with an Au/Pd layer using a Polaron E5100 coating system (Quorum Technologies, Laughton, United Kingdom).

### 2.4. Swelling Ratio (SR) and Water Retention (WR) of Cellulose-Based Hydrogels

The SR and WR of the cellulose-based hydrogels was evaluated under physiological conditions. Following synthesis, the hydrogels (8Cell, 14Cell, and 18Cell) were rinsed twice with Type 1 water and weighed (W_i_) after removing the surface excess water with a filter paper. Hydrogels were subsequently dried at RT for 24 h and weighed (W_d_). Then, hydrogel samples were incubated in phosphate-buffered saline (PBS) buffer under gentle agitation for 24 h at 37 °C and weighed again (W_s_). The SR and WR were calculated using Equations (1) and (2), respectively:

(1) $$\mathrm{SR}=\frac{W_{s}-{\;W}_{d}}{W_{d}}\times 100\%,$$

(2) $$\mathrm{WR}=\frac{W_{s}-{\;W}_{d}}{W_{i}-{\;W}_{d}}\times 100\%,$$

### 2.5. Analysis of the Degradation Profiles

Hydrogel degradation was also evaluated. First, hydrogel samples were rinsed with Type 1 water immediately after formation and weighed after removing the excess water from the surface with filter papers. The recorded values were taken as the total mass of the hydrogels. Afterwards, the hydrogels were incubated in PBS buffer, at 37 °C, under gentle shaking. Every two days, the mass loss was examined by weighing the samples, after eliminating the excess surface water. Fresh TST buffer was added to the system following each weighing. The degradation profile of the hydrogels was studied for 21 days.

### 2.6. Design, Production and Purification of CBM-Recombinant Protein Fusions

The fusion protein with an *N*-terminal green fluorescent protein (GFP) domain and CBM3 (GFP-CBM3) was purchased from NZYTech-Genes & Enzymes, Lisbon (5 mg/mL). The recombinant proteins ZZ-CBM3 and CBM3-Cys (CBM3C) were cloned in *Escherichia coli* (*E. coli*) by NZYTech. The CBM3-derived recombinant constructs used throughout this study are described in Table 2.

The fusion protein GFP-CBM3 was provided in 35 mM NaHepes buffer, pH 7.5, 750 mM NaCl, 200 mM imidazol, 3.5 mM CaCl_2_, and 3.2 M ammonium sulfate. Hence, to recover maximal GFP-CBM3 activity, a required volume of the precipitated protein suspension was centrifuged (13,000× *g* for 5 min) to remove the ammonium sulfate supernatant. The resulting pellet was resuspended in the same volume of 20 mM Tris-HCl, pH 7.5, 20 mM NaCl, and 5 mM CaCl_2_.

ZZ-CBM3 is a fusion of CBM3 with an N-terminal double Z-domain of protein A from *Staphylococcus aureus*. In this case, an insert totaling 861 base pairs was cloned into the NdeI-XhoI sites of a pET21a expression vector. The second recombinant protein, CBM3C, comprising a CBM3, a C-terminal cysteine, and an N-terminal histidine tag (6 amino acids), was constructed by inserting the coding sequences (552 base pairs) between the XhoI and NdeI sites of a pET28a expression vector (NZYTech). The peptide SSGPQQGLRANT was used to space CBM3C from the histidine tag. The full sequences of the CBM-based fusions are shown in Figure S1.

*E. coli* BL21 (DE3) cells were transformed with the resulting plasmids and grown in Luria–Bertani (LB) broth with the necessary selection marker (100 µg/mL ampicillin for pET-ZZCBM3-21a and 30 µg/mL kanamycin for pET-CBM3C-28a) at 37 °C and 250 rpm. Expression of the fusions was induced with 1 mM isopropyl β-D-1-thiogalactopyranoside (IPTG) as previously described [37]. Cells were harvested by centrifugation 3 h after induction and resuspended in a minimal volume of TST buffer or in a buffer containing 10 mM imidazole, 50 mM NaHEPES, 1 M NaCl, and 5 mM CaCl_2_, pH 7.5, for ZZ-CBM3-containing cells and CBM3C-containing cells, respectively.

Cells were sonicated (Bandelin Sonoplus) and the protein-rich supernatant was separated from debris by centrifugation. Then, ZZ-CBM3 was purified by affinity chromatography using a 1 mL column packed with IgG Sepharose 6 Fast Flow (GE Healthcare) connected to an ÄKTA 10 Purifier LC System (GE Healthcare). Unbound proteins were washed with TST buffer and bound ZZ-CBM3 was eluted with 0.5 M acetic acid, pH 2.8. After fraction collection, the pH is immediately neutralized with 3.2 M Tris buffer, pH 11 [38]. CBM3C was purified by Immobilized Metal Affinity Chromatography (IMAC) using a 1 mL nickel-containing HisTrap FF column (GE Healthcare) connected to an ÄKTA 10 Purifier LC System and an imidazole gradient [25]. The purified fusion proteins were stored at −20 °C before use.

The purity of ZZ-CBM3 and CBM3C was determined by sodium dodecyl sulfate–polyacrylamide gel electrophoresis (SDS-PAGE) using a NZYTech Low Molecular Weight (LMW) Protein Marker. Staining of the gel was performed with Coomassie Brilliant Blue, and images were obtained with a GS-800 Calibrated Densitometer (Figure S2). CBM3C protein samples were buffer exchanged to TST to remove excess imidazole using an Amicon^®^ Ultra-15 3k Centrifugal Filter Units, before determining the protein concentration. The concentration of the recombinant ZZ-CBM3 and CBM3C was estimated from the absorbance at 280 nm measured on a NanoDrop One Microvolume UV–Vis spectrophotometer (ThermoFisher, Waltham, MA, USA) using their molar extinction coefficient and molecular weight. The purified proteins were stored at −20 °C until required.

### 2.7. Binding of CBM Fusions to Cellulose Hydrogels

All binding experiments were made in TST buffer at 37 °C, under gentle agitation, using 1.5 mL polypropylene microcentrifuge tubes (unless stated otherwise). First, solutions of GFP-CBM3 with concentrations between 0.312 and 10.0 µM were prepared by diluting the 50 µM stock solution with TST buffer. In preparation for the binding experiments, approximately 5 mm discs of the hydrogels (8Cell, 14Cell, and 18Cell) weighing 30 mg were loaded onto 1.5 mL polypropylene microcentrifuge tubes and washed/equilibrated for 15 min with 100 µL of TST buffer. Then, 100 µL of solutions containing different concentrations (0.31–10 µM) of GFP-CBM3 (Table S1) were loaded into each well and incubated for 18 h at 37 °C, under gentle agitation. The same procedure was used to bind ZZ-CBM3 and CBM3C to the hydrogels (Table S1). After incubation, the samples were centrifuged at 10,000× *g* for 10 min at RT to remove any unbound protein and further washed three times with TST buffer. As controls, cellulose hydrogels were incubated with TST buffer without protein. The cellulose:GFP-CBM3, cellulose:ZZ-CBM3, and cellulose:CBM3C hydrogels and cellulose control were stored in TST buffer at 4 °C until further use.

### 2.8. Measuring the Binding of CBM-Based Fusions to the Hydrogels

The binding of GFP-CBM3 to the hydrogels was determined using a protocol directed to detect GFP fluorescence. Following contact of GFP-CBM3 with the hydrogels, supernatants were separated by centrifugation (2 min at 10,000× *g*) and transferred to the 96 wells of a white microplate. The fluorescence of the supernatants was measured using a Cary Eclipse fluorescence spectrophotometer (Varian) with excitation and emission set at 490 and 510 nm, respectively. The fluorescence of the GFP-CBM3 solutions prior to contacting the hydrogels was also measured in a microplate. Dilutions of CBM-based fusions were carefully adjusted, ensuring that measurements were performed in a region of linearity between fluorescence and concentration. To determine the binding of ZZ-CBM3 and CBM3C, the concentrations in solution before and after binding to the hydrogels were determined using a NanoDrop One Microvolume UV–Vis spectrophotometer by measuring the absorbance at 280 nm and using their molar extinction coefficient and molecular weight.

Mass balance calculations were then performed using the initial concentration of CBM ([CBM]_Initial_) and the concentration of CBM in supernatants after incubation with hydrogels ([CBM]_Free_, nM) to determine the equilibrium concentrations of GFP-CBM3, ZZ-CBM3, and CBM3C in the solid ([CBM]_Bound_, nmol·g^−1^) phase (Equation (3)).

(3) $${\left[\mathrm{CBM}\right]}_{\mathrm{Bound}}=\frac{\left({\left[\mathrm{CBM}\right]}_{\mathrm{Initial}}-{\left[\mathrm{CBM}\right]}_{\mathrm{Free}}\right)V}{\mathrm{cellulose}\;\mathrm{weight}}$$

Each binding condition was tested in triplicate. The binding data were analyzed using nonlinear regression analysis and a Langmuir independent binding site(s) model (Equation (4)).

(4) $${\left[\mathrm{CBM}\right]}_{\mathrm{Bound}}=\frac{B_{\max}\;{\left[\mathrm{CBM}\right]}_{\mathrm{Free}}}{K_{d}+{\left[\mathrm{CBM}\right]}_{\mathrm{Free}}},$$

where B_max_ (nmol·g^−1^) is the maximum binding capacity of cellulose hydrogels and K_d_ (nM) is the equilibrium dissociation constant of the cellulose:CBM complex.

The binding of the GFP-CBM3 fusion was also analyzed qualitatively by examining the hydrogels recovered after incubation by fluorescence microscopy (Leica DMLB; Leica Microsystems, Wetzlar, Germany). Control experiments were performed using GFP alone.

### 2.9. Storage Stability of Immobilized Recombinant CBM Fusion Proteins

The three cellulose-based hydrogels were incubated with the CBMs as described (Section 2.7). Non-immobilized CBM was removed by washing cellulose samples and controls three times with TST buffer at 10,000× *g* for 5 min. Afterwards, the hydrogels were pelleted by centrifugation (10 min at 10,000× *g*), the supernatant was withdrawn, and the hydrogels pellets were resuspended with 200 µL fresh TST buffer and incubated at 4 °C for 15 days. After incubation, cellulose-bound and free CBM were separated by centrifugation (2 min at 10,000× *g*), and the concentration of free CBM in supernatant was measured as described (Section 2.8). Controls were prepared by incubating hydrogels with TST buffer in the same reaction conditions. This experiment was done in triplicate.

### 2.10. Testing of the Functionality of CBM-X Fusions

The ability of the CBM3-based fusions to bind and anchor their counterpart biomolecule onto cellulose was tested by performing pull-down assays with the modified hydrogels. The assays involved the capture (Figure 1B) or covalent binding (Figure 1C) of the respective counterpart from solution by hydrogels pre-modified with the CBM fusions and washing of unbound molecules.

#### 2.10.1. ZZ-CBM3

The functionality of the cellulose:ZZ-CBM3 complexes was evaluated by capturing the immunoglobulin G (IgG) antibody from solution, washing, and quantifying the binding efficacy with a NanoDrop. A total of 100 μL of a solution of IgG in TST buffer was added and incubated for 18 h at 37 °C with the ZZ-CBM3-modified hydrogels. Controls were performed by incubating IgG with unmodified hydrogels. The suspension was then centrifuged (10,000× *g* for 10 min) and the supernatant was withdrawn. The absorbance at 280 nm was recorded using a NanoDrop and the binding was calculated by mass balance as described for CBM capture (Equation (3)). Functionalization and control experiments were performed in triplicate.

#### 2.10.2. CBM3C

The functionality of cellulose:CBM3C complexes was studied by promoting the covalent binding of DN3, an amino-serinol-modified oligonucleotide to the terminal cysteine of CBM3C (Figure S3). As a first step, the hetero-bifunctional crosslinker sSMCC, which contains N-hydroxysuccinimide (NHS) ester and maleimide groups, was added to the amino-serinol terminus of the oligo as follows. The DN3 oligo was dissolved in phosphate buffer (100 mM sodium phosphate, 100 mM NaCl, pH 7.3) to a final concentration of 100 μM, and 10 mM sSMCC was added to 50 μL of DNA solution in a 50-fold molar excess. The reaction mixture was incubated for 30 min at RT, and the excess crosslinker was removed with a Micro Bio-Spin^TM^ 6 column (Bio Rad) equilibrated with phosphate buffer. Samples were stored at −20 °C if not used immediately. Pre-functionalized cellulose:CBM3C discs of hydrogel were loaded onto 1.5 mL polypropylene microcentrifuge tubes. The modified maleimide-DN3 oligo was then incubated overnight at RT with CBM3C at a 3:2 molar ratio ([DNA]:[CBM3C]). Next, the hydrogels were separated by centrifugation, and the concentration of free DN3 in supernatant was measured at 260 nm with a Nanodrop spectrophotometer. These tests were repeated in triplicate. For comparison purposes, DN3 was incubated with unmodified hydrogels, and cellulose:CBM3C hydrogels were incubated with TST buffer without DNA as control.

### 2.11. Statistical Analysis

Experiments were repeated three times, and the results were expressed as a mean ± standard deviation. Statistical significance was calculated using the repeated measures one-way analysis of variance (ANOVA) with Tukey’s multiple comparison test (swelling and water retention studies), two-way ANOVA with Tukey’s multiple comparison test (degradation studies), and Student’s *t*-test (immobilization studies). A comparison between two means was analyzed with statistical significance level set at *p* < 0.05.

## 3. Results and Discussion

### 3.1. Surface Morphology Analysis

**Figure 2:**
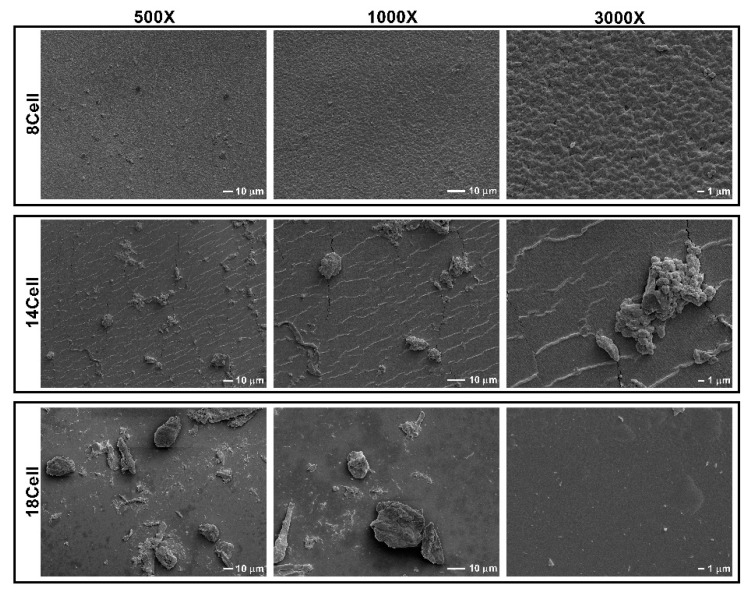
Scanning electron microphotographs of cellulose-based hydrogels synthesized with 8, 14 and 18% wt. MCC (8Cell, 14Cell and 18Cell, respectively). SEM images of the different cellulose hydrogels tested at different magnifications were obtained: 500× (scale bar: 10 µm), 1000× (scale bar: 10 µm), and 3000× magnification (scale bar: 1 µm).

The surface of the hydrogels was investigated using SEM imaging (Figure 2). All hydrogels are characterized by a rugged and compact structure. The hydrogel prepared with the lowest cellulose concentration (8Cell) displayed the roughest surface but also a more uniform network. On the contrary, the structure of 14Cell and 18Cell hydrogels varied considerably. Further, aggregates of various sizes were observed on their surfaces, a phenomenon that suggests that partial phase separation occurred during crosslinking. Indeed, synthesizing the 14Cell and 18Cell hydrogels with a smooth surface was not easy to accomplish, as the higher amounts of MCC required were more difficult to dissolve. This may lead to the observed aggregation of MCC throughout the hydrogel matrix. The images further show that hydrogels prepared with higher amounts of cellulose are denser, with 18Cell hydrogels displaying the most compact network.

A recent report described the microstructure of equivalent cellulose-based hydrogels prepared using an IL/DMSO mixed solution [21]. Briefly, a porous three-dimensional network structure was formed at a wide range of cellulose concentrations. Hydrogels with cellulose concentrations between 8 and 18 wt.% yielded network structures with average pore diameters of 3.08 ± 0.17 and 0.13 ± 0.01 μm, respectively. In this context, the microstructure became denser as the cellulose concentration increased due to a reduction in the distance between cellulose chains. These findings are also associated with an increased hydrogen-bonding interaction between cellulose chains, which led to the enhancement of the mechanical properties of the hydrogels [21].

### 3.2. Swelling and Water Retention Studies

**Figure 3:**
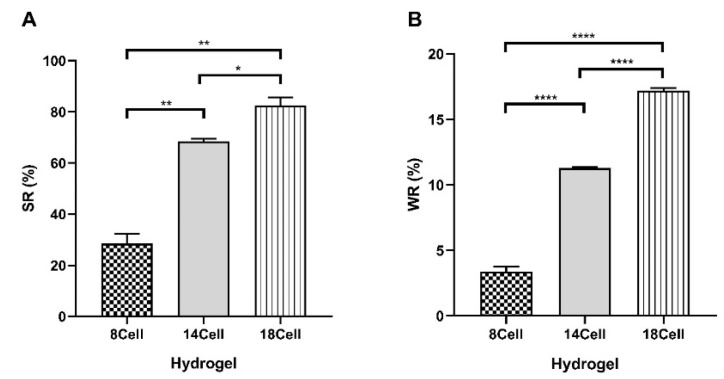
(**A**) SR and (**B**) WR of hydrogels synthesized with 8, 14 and 18% wt. cellulose (8Cell, 14Cell, and 18Cell, respectively) obtained after 24 h incubation in PBS buffer. Statistical analysis performed with one-way ANOVA analysis. Statistic difference is marked with * (*p* < 0.05), ** (*p* < 0.01), or **** (*p* < 0.0001). Data represent mean ± standard deviation (SD).

The SR and WR are two key parameters used to characterize a hydrogel network structure. De-swelling and swelling tests (Table S2) were performed to determine the effect of cellulose concentration on the SR (Figure 3A) and WR (Figure 3B) of the hydrogels.

Overall, increasing the cellulose content improved the SR and water retention capacity of the hydrogels. The hydrogel with the lowest MCC concentration (8Cell) quickly lost most of its water content (approximately 89% of the hydrogel mass) by evaporation (Table S2). Interestingly, higher water retention capacity was observed for 14Cell and 18Cell hydrogels, namely 11.3% and 17.2%, respectively. This might be due to the increase of physical cross-linking and reduced porosity of these hydrogels that results from a more extensive hydrogen bonding formation between water molecules and the larger number of functional groups of the hydrogel matrix. This enhanced hydrogen bond network delayed the evaporation of the absorbed water molecules and thus improved water retention capacity. In a recent study, a similar phenomenon was reported in rice husk ash-based superabsorbent hydrogels [43]. The mechanical properties of similar cellulose-based hydrogels were evaluated through compressive strength and modulus and fracture energy in a previous report by Satani et al. [21]. Compressive strength and modulus and fracture energy were proportional to the cellulose concentration. Hence, those results support the notion that an increase in cellulose concentration enhanced the interactions between cellulose chains, namely hydrogen bonding and physical entanglement [21].

Swelling properties of the hydrogels were analyzed following 24 h of incubation in PBS buffer, a commonly used swelling medium [44]. The swollen hydrogels did not fully recover their original dimensions after soaking in water. Hydroxyl groups in cellulose chains come closer during hydrogel shrinkage and form a dense hydrogen network in the solid, which prevents swelling after soaking the dried solids in water. However, the cellulose content strongly affected the SR. While all materials showed relatively low SR values, a significant increase of SR was observed with an increase in the cellulose content (*p* < 0.05; one-way ANOVA). In this case, the higher SR can be associated with the remnant MCC aggregates visible in the SEM micrographs of Figure 2. In these hydrogels, the SR slightly increases with increase in cellulose concentration. The ability to retain water is strongly dependent on both the hydrophilicity and morphology of the material. Since all compounds used in the synthesis are hydrophilic, the differences in SR observed most likely resulted from differences in the morphology of the hydrogels. The results of swelling experiments clearly show that the hydrogels are able to absorb a significant amount of water, and hence that they are likely to mimic the natural aqueous surroundings of cells very well [45].

### 3.3. Degradation Profile of the Hydrogels

**Figure 4:**
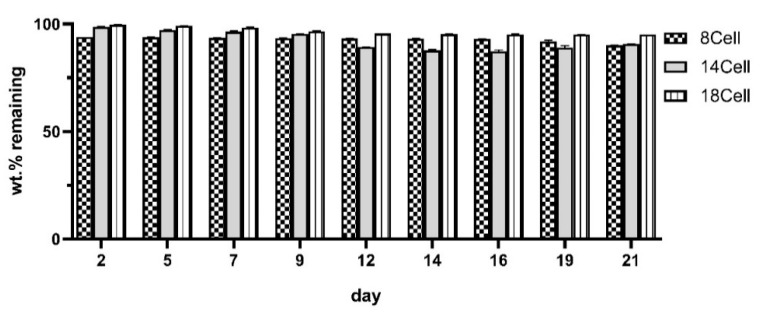
Degradation of the hydrogels during incubation in PBS buffer. The variation of hydrogel weight during the course of 21 days of incubation at 37 °C is shown for the 8Cell, 14Cell, and 18Cell hydrogels.

The degradation of the hydrogels during incubation in PBS buffer at 37 °C for 21 days was studied. The results depicted in Figure 4 show the remaining weight of hydrogels (wt.%) at the defined time of the experiment. In general, the largest weight loss was observed during the first 12 days (~7%, ~11%, and ~4% for 8Cell, 14Cell, and 18Cell, respectively). From then on, changes were not substantial, with weight stabilizing at various levels, depending on the type of hydrogels. The weight of the 18Cell hydrogels stabilized at ~95%, whereas, for materials with lower cellulose concentration (8Cell), the weight stabilized at ~90%. The weight loss is lower in the case of the materials with the highest cellulose content, most likely due to the more rigid and stable structure of the hydrogel that can hold water more efficiently [46,47]. This result is in accordance with the abovementioned higher water retention capacity of the 18Cell hydrogel.

### 3.4. Recombinant CBM Fusion Proteins

Recombinant CBM3-based fusion proteins were chosen as model fusion tags in this work. CBM3 from the cellulosomal-scaffolding protein A (CipA) is responsible for the structural organization of the cellulosomes present in *C. thermocellum* and is well-known for enabling facile and strong binding to cellulosic materials [35]. Previous studies using paper fibers demonstrated that CBM3 binding to cellulose is very stable. Nevertheless, while it is very difficult to remove bound CBM3, a gradual transfer between fibers was observed [48]. Recombinant expression of bi-functional ZZ-CBM3 as well as CBM3C were achieved using the pET21a and pET28a expression system, respectively, in *E. coli* BL21(DE3) cells. Both recombinant proteins were successfully expressed. Afterwards, the proteins were purified by IMAC (CBM3C) and affinity chromatography (ZZ-CBM3), and estimated molecular mass was confirmed through SDS-PAGE. As shown in Figure S2, the purified soluble fraction of ZZ-CBM3 and CBM3C recombinant proteins exhibited the expected MW (31.9 and 20.2 kDa, respectively).

### 3.5. Binding of the CBM Fusions to the Hydrogels

**Figure 5:**
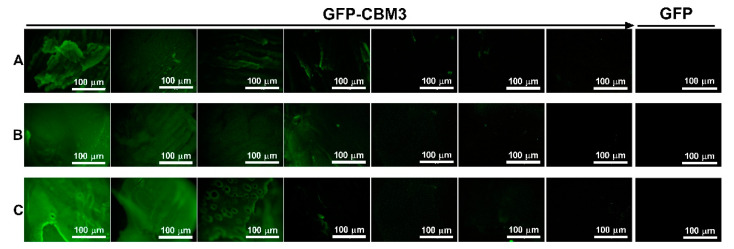
Binding of GFP-CBM3 fusions to cellulose hydrogels. Fluorescence microcopy images of 8Cell (**A**), 14Cell (**B**), and 18Cell (**C**) hydrogels following incubation with solutions of decreasing GFP-CBM3 concentration (from 10.0 to 0.312 µM), as indicated by the arrow. Control experiments were performed by incubating hydrogels with 10 µM of GFP (right column). Images were taken with a fluorescence microscope (magnification, 4×; scale bar, 100 µm).

The binding of CBMs towards the synthesized cellulose-based hydrogels was studied (Figure 1). Initial qualitative experiments compared the ability of a GFP-CBM3 fusion and a GFP control to bind to the hydrogels. Fluorescence microscopy data (Figure 5) show that fluorescence was only visible when GFP was fused to CBM3. This suggests that CBM3 in the fusion is fully functional and binds effectively to hydrogels.

**Figure 6:**
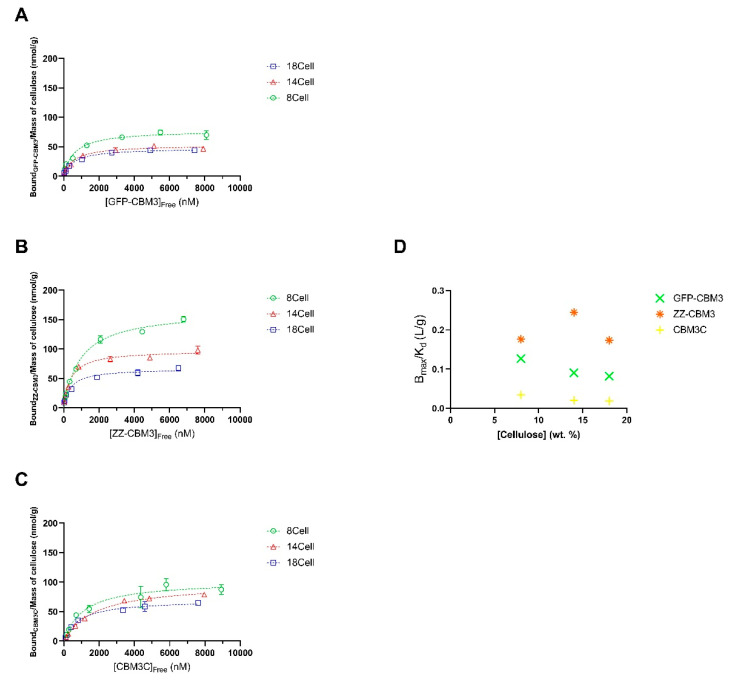
Binding isotherms of (**A**) GFP-CBM3, (**B**) ZZ-CBM3, and (**C**) CBM3C depends on cellulose concentration. Solid lines represent best fits of a Langmuir one binding site model. Error bars are from at least three independent measurements. (**D**) B_max_/K_d_ values corresponding to the binding potential of GFP-CBM3 (in green), ZZ-CBM3 (orange), and CBM3C (yellow) at different MCC concentrations.

CBM:hydrogel binding isotherms were then determined for GFP-CBM3, ZZ-CBM3, and CBM3C using 100 mM phosphate buffer, pH 7.0, at 37 °C (Figure 6A–C). In general, the isotherms followed the traditional logarithmic-like curve of the Langmuir binding model. For the range of CBM protein loads tested (up to ~74 nmol per g of MCC present in the hydrogel), the maximal binding capacities (B_max_) in the solid phase at equilibrium (Table 3) for 8Cell, 14Cell, and 18Cell were ~78–165, ~53–99, and ~48–70 nmol/g, respectively. As shown in Figure 6, more efficient binding was observed at lower cellulose concentrations. Further, B_max_ and K_d_ values were highly dependent on cellulose concentration and the specific CBM protein under study (Table 3). A plot of B_max_/K_d_ (binding potential) as a function of cellulose concentration revealed an inverse relationship (Figure 6D), which confirms that highest cellulose concentration is associated with lower binding potential. B_max_/K_d_ is defined by the initial slope of the isotherms and is less impacted by higher [CBM]_Free_ concentrations explored in our study. However, Figure 6D shows a maximum binding potential for ZZ-CBM3 in 14Cell hydrogel. In this context, further studies are required to thoroughly characterize the binding interactions between ZZ-CBM3 and cellulose hydrogels, for instance, using a fluorescence-labeled ZZ-CBM3, and a thorough analysis of their binding capacity under different reaction conditions, such as pH and temperature, should also be performed.

Interestingly, the binding isotherms of cellulases are assumed to be independent of cellulose concentration. This assumption relies on the fact that the number of binding sites present per gram of cellulose and their binding affinity are independent of cellulose concentration. Several studies have already focused on the analysis of binding isotherms of cellulases measured almost exclusively at one cellulose concentration. However, Wang et al. [49] found that the binding of crude cellulase was stronger at lower Avicel concentrations. They hypothesized that higher cellulose concentrations caused the association of cellulose microfibrils, thus leading to a reduction in specific surface area accessible for binding [49]. In a different study, it was suggested that the decrease in the surface area associated with increasing cellulose concentration was accountable for lower association rate constants between cellulose and cellulases registered at higher cellulose concentrations [50].

Consistent with the binding data herein described, a SEM study performed by Kuijk et al. revealed that the formation of large aggregates in a bacterial cellulose (BC) suspension is dependent on the BC concentration [51]. As described above, the assembly of BC microfibrils is expected to decrease the available surface area for the binding of cellulases. Accordingly, B_max_ should decrease with increasing cellulose concentration, whereas K_d_ is expected to remain unaltered. However, the precise mechanism of the reduced binding efficiency of the CBMs with increasing cellulose concentration in the hydrogels remains to be studied. Nevertheless, the B_max_/K_d_ data presented in Figure 6D support the suggestion that increasing the cellulose concentration may cause the association of cellulose microfibrils within the hydrogel network, which results in a decrease in surface area available for the binding of CBM [52].

### 3.6. Storage Stability of Immobilized CBM Fusion Proteins

Storage stability of the immobilized protein was tested by storing the modified hydrogels in PBS buffer for 15 days at RT and 4 °C followed by determination of the residual protein concentration in the supernatants. The protein-modified hydrogels exhibited good stability when stored at both RT and 4 °C as no protein was detected in the supernatant by either fluorescence analysis (GFP-CBM3) or NanoDrop quantification (ZZ-CBM3 and CBM3C). This result suggests that cellulose-based hydrogels were able to maintain a high protein retention.

### 3.7. Testing of the Functionality of CBM-X Fusions

**Figure 7:**
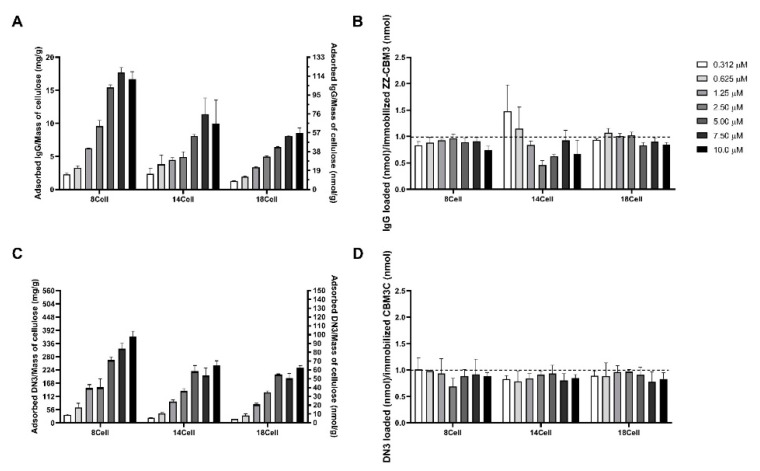
Binding of IgG and DN3 oligonucleotide onto CBM3-modified cellulose. Grafting of (**A**) IgG to hydrogels previously modified with ZZ-CBM3 and (**C**) of DN3 to hydrogels previously modified with CBM3C. Molar ratio of grafted (**B**) IgG and (**D**) DN3 to immobilized ZZ-CBM3 and CBM3C, respectively. Discs of CBM3-modified hydrogels (5 mm, 30 mg) were contacted with 0.312–10.0 µM of IgG or DN3 oligonucleotide. The concentration of IgG or DN3 in the liquid phase at equilibrium with the materials was determined with a NanoDrop spectrophotometer and used to calculate the amount of IgG or DN3 in the solid phase by performing a mass balance. The dashed horizontal lines represent 1:1 binding model between IgG and ZZ-CBM3, and DN3 and CBM3C under the experimental conditions used. Error bars were obtained from the SD of three measurements.

Hydrogels pre-modified with different amounts of ZZ-CBM3 and CBM3C were exposed to IgG and DN3 solutions to promote capture of IgG and covalent binding of DN3, respectively (see Figure 1B,C, respectively). The determination of IgG and DN3 density in the resulting materials shows that both biomolecules were effectively grafted onto the hydrogels (Figure 7). Figure 7A shows that the use of increased amounts of ZZ-CBM3 in the hydrogels increased the amount of IgG that could be captured. These results clearly indicate that biochemical coupling via ZZ-CBM3 is a practicable strategy to immobilize antibodies on cellulose hydrogels. A similar trend was observed for the covalent binding of oligo DN3 to CBM3C (Figure 7C).

The average molecular densities of immobilized IgG and DN3 were similar. In the case of IgG (Figure 7A), the density ranged from ~15.0 ± 1.16 to ~110 ± 7.97, ~15.8 ± 5.44 to ~66.2 ± 24.2, and ~8.67 ± 0.306 to ~56.8 ± 5.09 nmol/g, for 8Cell, 14Cell, and 18Cell, respectively. Densities of ~9.13 ± 0.416 to ~97.1 ± 7.03, ~5.77 ± 0.208 to ~64.0 ± 6.50, and ~4.40 ± 0.100 to ~61.6 ± 3.16 nmol/g were obtained for DN3 immobilization (Figure 7C). This corresponds to biomolecule grafting yields relative to the initial amounts contacted with the CBM3-modified hydrogels that vary between ~26% and ~89% for IgG and ~14% and ~85% for DN3.

Significant amounts of either biomolecule were bound to the hydrogels (Figure 7A,C). A comparison of Figure 7A,C also shows that the amount of bound molecule was higher in materials that had more ZZ-CBM3 or CBM3C. The molar ratio of grafted IgG or DN3 to immobilized ZZ-CBM3 or CBM3C was close to 1 for all hydrogels except for the immobilization of lower IgG concentrations, namely 0.312 and 0.625 µM, onto the 14Cell hydrogel (Figure 7B,D). In both cases, the expected molar ratio of 1:1 binding of the small ZZ-CBM3 fusion to IgG was not observed. This suggests that more than one IgG molecule bonded to ZZ-CBMs. Further tests would be required to assess the binding ratio between ZZ-CBMs and IgG antibodies and confirm this hypothesis.

Nonetheless, these findings closely agree with the results of a previous study conducted by Ljungquist et al., in which the double Z-domain recombinant receptor based on staphylococcal protein A could bind two IgG molecules [53]. In another study, elastin-like-protein (ELP) was fused to Z domains for the purification and recovery of antibodies, and the authors concluded that ELP-ZZ presented a higher binding affinity to IgG than ELP-Z [54]. In addition, it was shown very recently that the ZZ-domain can bind to two IgG molecules when used as an affinity ligand for antibody purification [55]. Regarding higher IgG concentrations, the ZZ-CBM3:IgG binding ratio was less than 1:1. Considering the higher ZZ-CBM3 surface density, when the IgG molecule was attached by one ZZ-CBM3 molecule, the surrounding ZZ-CBM3 molecules were covered by the Fab arms, limiting their accessibility to other IgG molecules. In fact, a previous study has shown that, at high receptor densities, larger analyte molecules can crowd the surface and hinder further analyte binding [56]. However, further studies are required to thoroughly characterize the binding interactions between ZZ-CBM3 and IgG molecules, for instance, using a fluorescence-labeled ZZ-CBM3.

Taken together, these results are consistent with the successful binding of IgG or DN3 to ZZ-CBM3 or CBM3C, respectively. This shows that not only were CBM3 fusions successfully immobilized on the cellulose hydrogels but also that those fusions maintained their ability to bind their bioactive counterpart.

## 4. Conclusions

We demonstrated that biomolecular recognition is a successful approach for the functionalization of cellulose-based hydrogels with a fluorescent protein, a protein domain that recognizes IgG antibodies, and a C-terminal cysteine to covalently link DNA strands. Noteworthy, the results suggest that these CBM3-based fusions were able to efficiently bind cellulose even if it is present in a hydrogel form.

Regarding the fluorescent protein, GFP-CBM3 was immobilized on the hydrogels and GFP retained their fluorescence. Additionally, the effective anchoring of IgG and DN3 on cellulose hydrogels via the bound bi-functional fusion tags ZZ-CBM3 and CBM3C, respectively, demonstrated that the two CBM3 fusions remained active after immobilization and were able to capture and bind their expected target.

This proof-of-concept study demonstrates that bi-functional fusion tags of CBM3 can serve as a useful handle for anchoring bioactive biomolecules to cellulosic hydrogel materials. This approach can be applied to other biomolecules and could form the basis of a highly versatile platform for the development of cellulose-based materials, including hydrogels, with multiple biomedical applications.

## Figures and Tables

**Table 1:** Composition of solutions used to prepare hydrogels with different cellulose concentrations.

Entry	Cellulose (g)	[EMIM][Ac] (g)	DMSO (g)	[Cellulose] (wt.%) ^1^	Sample
1	0.4	3	2	8	8Cell
2	0.7			14	14Cell
3	0.9			18	18Cell

^1^ Cellulose weight percent is based on the total weight of the solvents.

**Table 2:** Recombinant bi-functional CBM3 fusion proteins used in this work. Data for the native CBM3 protein are provided for comparison.

Fusion Protein	Number of Amino Acids	MW (KDa)	Extinction Coefficient, ε (M^−1^·cm^−1^)
CBM3	159	17.6	35,410
CBM3 derivatives			
GFP-CBM3	574	63.2	73,020
ZZ-CBM3	286	31.9	38,390
CBM3C	184	20.2	35,535

**Table 3:** Maximum binding capacities, B_max_, and dissociation constants, K_d_, for CBMs on cellulose hydrogels obtained from the data shown in Figure 6. The partitioning coefficients (B_max_/K_d_) for CBMs were obtained from the Langmuir isotherm fitted to the data in Figure 6.

Parameters	8Cell			14Cell			18Cell		
	GFP-CBM3	ZZ-CBM3	CBM3C	GFP-CBM3	ZZ-CBM3	CBM3C	GFP-CBM3	ZZ-CBM3	CBM3C
B_max_ (nmol/g)	77.81	165.3	102.1	52.77	97.71	98.55	47.80	66.59	69.88
K_d_ (nM)	599.7	940.4	1113	566.0	400	1769	586.3	383.9	853.6
B_max_/K_d_ (L/g)	0.1297	0.1758	0.09173	0.09323	0.2443	0.05571	0.08153	0.1735	0.08187

## Data Availability

The data that support the findings of this study are available from the corresponding author, D.M.F.P., upon reasonable request.

## Supplementary Materials

The following are available online at https://www.mdpi.com/article/10.3390/ma14123175/s1, Figure S1: Amino acid sequence of the proteins (A) GFP-CBM3, (B) ZZ-CBM3 and (C) CBM3C., Figure S2: SDS-PAGE analysis of CBM3 proteins and fusions, whether purified or commercially purchased, Table S1: Initial protein concentrations used for immobilization onto cellulose-based hydrogels surface, Figure S3: Two-step reaction scheme for conjugating cellulose:CBM3C complexes and an oligonucleotide with sSMCC, Table S2: Overview of de-swelling and swelling tests used to determine the SR and WR of the three hydrogels studied.
