# Supplementary material for: Functionalization of Cellulose-Based Hydrogels with Bi-Functional Fusion Proteins Containing Carbohydrate-Binding Modules
Source: Materials (Basel). 2021 Jun 9;14(12):3175. doi: 10.3390/ma14123175 (PMC8227779; doi:10.3390/ma14123175)
Supplement: Supplementary file 1 [file materials-14-03175-s001.zip › materials-1231568-supplementary.pdf]

materials

Supplementary Material

Functionalization of Cellulose-Based Hydrogels with Bi-Functional Fusion Proteins Containing Carbohydrate-Binding Modules

Design, production, and purification of ZZ-CBM3 and CBM-C

The bi-modular recombinant fusions ZZ-CBM3 (31.9 kDa) and CBM3C (20.2 kDa) were designed with the following features. The ZZ-CBM3 fusion protein combines an N-terminal double Z domain derived from the staphylococcal protein A, a 10 amino acid (aa) long linker and a C-terminal CBM – CBM3 from *C. thermocellum* ATCC 27405. CBM3C recombinant protein, comprised CBM3, a C-terminal cysteine and an N-terminal histidine tag (6 amino acids).

The amino acid sequence of each resulting protein is displayed in **Error! Reference source not found.**. The molecular weights of constructs indicated above were calculated with the ExPASy ProtParam tool.

(A) GFP-CBM3 (N→C)

574 amino acids
MW: 63.2 KDa

MSDKIIHLTDDSFDTDVLKADGAILVDFWAEWCGPCKMIAPILDEIADEYQGKLTVAKLNIDQNPGTAPKYGIR
GIPTLLLFKNGEVAATKVGALSKGQLKEFLDANLAGSGSGHM**HHHHHH**SSGLVPRGSGMKETAAAKFERQ
HMDSPDLGTDDDDKAMGYLGSEFVSKGEELFTGVVPILVELDGDVNGHKFSVSGEGEGDATYGKLTLKFI
CTTGKLPVPWPTLVTTLTYGVQCFARYPDHMKQHDFFKSAMPEGYVQERTIFFKDDGNYKTRAEVKFEG
DTLVNRIELKGIDFKEDGNILGHKLEYNYNSHKVYITADKQKNGIKVNFKTRHNIEDGSVQLADHYQQNTPI
GDGPVLLPDNHYLSTQSALSKDPNEKRDHMVLLEFVTAAGITLGMDELYKSSGLVPRGSTPVSGNLKVEFY
NSNPSDTTNSINPQFKVTNTGSSAIDLSKLTLRYYYTVDGQKDQTFWCDHAAIIGSNGSYNGITSNVKGTF
VKMSSSTNNADTYLEISFTGGTLEPGAHVQIQGRFAKNDWSNYTQSNDYSFKSASQFVEWDQVTAYLNG
VLVWGKEP

(B) ZZ-CBM3 (N→C)

285 amino acids
MW: 31.9 KDa

MDNKFNKEQQNAFYEILHLPNLNEEQRNAFIQSLKDDPSQSANLLAEAKKLNDAQAPKVDNKFNKEQQNA
FYEILHLPNLNEEQRNAFIQSLKDDPSQSANLLAEAKKLNDAQAPKVSSGLVPRGSTPVSGNLKVEFYNSN
PSDTTNSINPQFKVTNTGSSAIDLSKLTLRYYYTVDGQKDQTFWCDHAAIIGSNGSYNGITSNVKGTFVKMS
SSTNNADTYLEISFTGGTLEPGAHVQIQGRFAKNDWSNYTQSNDYSFKSASQFVEWDQVTAYLNGVLVW
GKEP

(C) CBM3C (N→C)

183 amino acids
MW: 20.2 KDa

MGSS**HHHHHH**SSGPQQGLRANTPVSGNLKVEFYNSNPSDTTNSINPQFKVTNTGSSAIDLSKLTLRYYYT
VDGQKDQTFWCDHAAIIGSNGSYNGITSNVKGTFVKMSSSTNNADTYLEISFTGGTLEPGAHVQIQGRFA
KNDWSNYTQSNDYSFKSASQFVEWDQVTAYLNGVLVWGKEPGGC

Figure S1. Amino acid sequence of the proteins (A) GFP-CBM3, (B) ZZ-CBM3 and (C) CBM3C. Green—GFP protein; orange—linker; blue—CBM3; brown—ZZ domain; bold—His tag; red—C-terminal cysteine.

Following purification, the eluted recombinant proteins were run on an SDS-PAGE gel (Figure S2), with the objective of assessing presence and purity of said proteins. The first lane, labeled M, correspond to the NZYTech Low Molecular Weight Protein Marker, with proteins ranging from 18.5 to 96 kDa. Lane 1 contains commercial CBM3 (for comparison purposes only), with a molecular weight of 19.6 kDa; Lanes 2 and 3 correspond to the purified CBM3C and ZZ-CBM3 samples, respectively. Lane 4 shows the commercial GFP-CBM3 construct (63.2 kDa). Only a single band is observed in the lanes with the purified proteins, which attests to their high purity. All CBM proteins, purified and purchased, show an accurate migration in the gel, according to the molecular weight marker. Thus, it is safe to assume that the production of ZZ-CBM3 and CBM3C was successful.

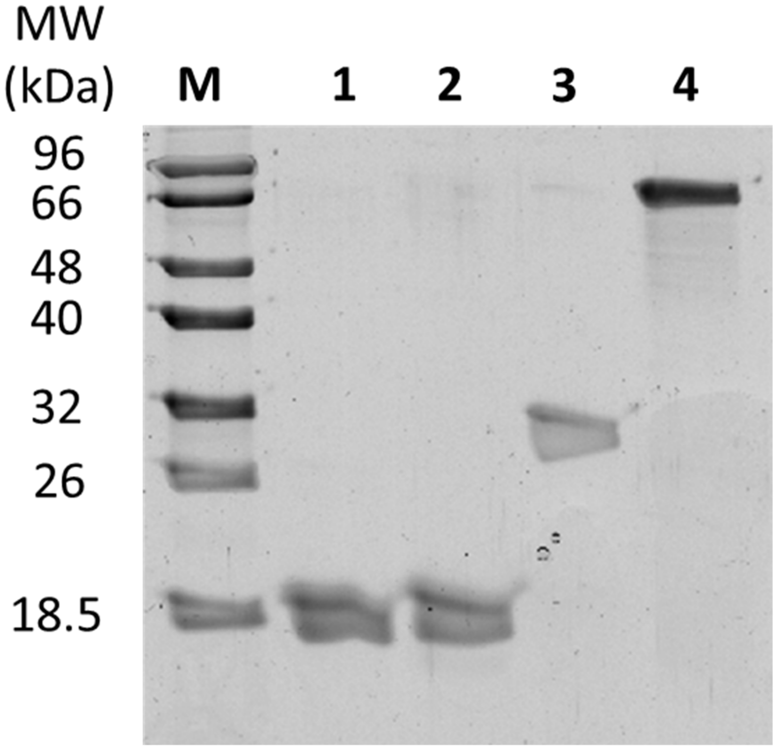

Figure S2. SDS-PAGE analysis of CBM3 proteins and fusions, whether purified or commercially purchased. M – NZYTech Low Molecular Weight (LMW) Protein Marker; 1—CBM3 (19.6 kDa); 2—CBM3C (20.2 kDa); 3—ZZ-CBM3 (31.9 kDa); 4—GFP-CBM3 (63.2 kDa). Purified samples correspond to lanes 2 and 3. Lanes 1 and 4 correspond to commercially purchased protein samples.

Table S1. Initial protein concentrations used for immobilization onto cellulose-based hydrogels surface.

$[\text{Protein}]_{\text{initial}}$ (μM)	$[\text{GFP-CBM3}]_{\text{initial}}$ (mg/mL)	$[\text{ZZ-CBM3}]_{\text{initial}}$ (mg/mL)	$[\text{CBM3C}]_{\text{initial}}$ (mg/mL)
10.0	0.574	0.319	0.202
7.50	0.431	0.239	0.151
5.00	0.287	0.159	0.101
2.50	0.144	0,0797	0,0504
1.25	0,0718	0,0398	0,0252
0.625	0,0359	0,0199	0,0126
0.312	0,0179	0,00996	0,00630

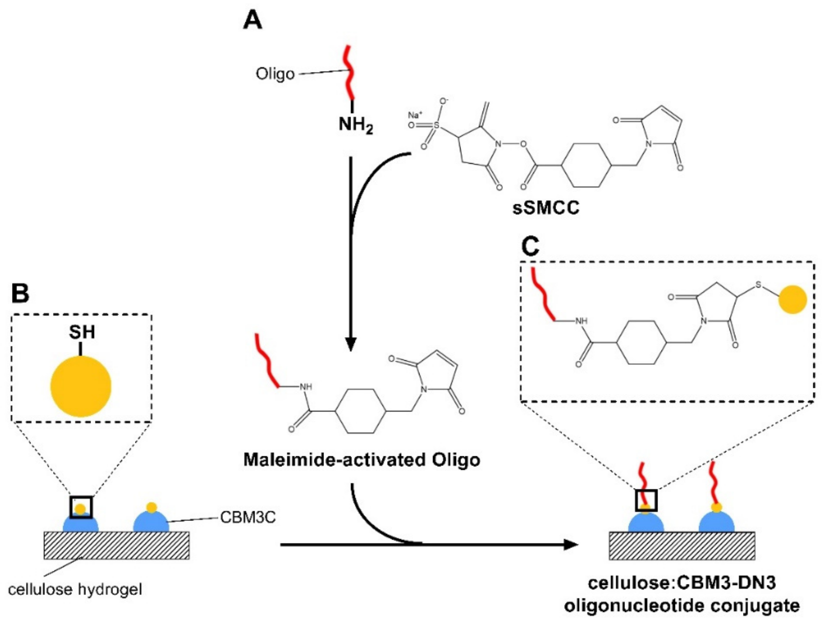

Figure S3. Two-step reaction scheme for conjugating cellulose: CBM3C complexes and an oligonucleotide with sSMCC. Briefly, (A) the crosslinker is first reacted with the amino-serinol-modified oligonucleotide DN3 to produce a maleimide-activated oligonucleotide. After excess non-reacted crosslinker and by-products are removed, (B) the maleimide-activated oligonucleotide is reacted with cellulose: CBM3C complexes having sulfhydryl groups, yielding (C) cellulose: CBM3-DN3 oligonucleotide conjugates.

Table S2. Overview of de-swelling and swelling tests used to determine the SR and WR of the three hydrogels studied.

		Hydrogel code		
		**8Cell**	**14Cell**	**18Cell**
**Hydrated**	W_i (mg)	87.1	87.2	87.2
**Dry**	W_d (mg)	9.27	12.3	15.1
	Mass loss	89.4%	85.8%	82.7%
**Re-hydrated**	W_s (mg)	11.9	20.8	27.5
	SR	28.5%	68.4%	82.6%
	WR	3.38%	11.3%	17.2%
